# Living with Reduced Income: An Analysis of Household Financial Vulnerability Under COVID-19

**DOI:** 10.1007/s11205-021-02811-7

**Published:** 2021-10-08

**Authors:** Catarina Midões, Mateo Seré

**Affiliations:** 1 Ca’ Foscari University of Venice, Venice, Italy; 2 Bruegel, Brussels, Belgium; 3 University of Antwerp, Antwerp, Belgium; 4 Pompeu Fabra University, Barcelona, Spain

**Keywords:** Vulnerability, Household finance, Microsimulation, COVID-19

## Abstract

The COVID-19 crisis has led to substantial reductions in earnings. We propose a new measure of financial vulnerability, computable through survey data, to determine whether households can withstand a certain income shock for a defined period of time. Using data from the ECB Household Finance and Consumption Survey (HFCS) we analyse financial vulnerability in seven EU countries. We find that, out of the 243 million individuals considered, 47 million are vulnerable to a three month long income shock (the average length of the first wave COVID-19 lockdown), i.e., they cannot afford food and housing expenses for three months without privately earned income. Differences across countries are stark. Individuals born outside the EU are especially likely to be vulnerable. Being younger, a single parent, and a woman are also statistically significant risk factors. Through a tax-benefit microsimulation exercise, we look into the COVID-19 employment protection benefits, the largest income support measure in the countries considered. Considering as our sample individuals in households where someone receives a salary, we derive household net income when employees are laid-off and awarded the COVID-19 employment protection benefits enacted. Our findings suggest that the employment protection schemes are extremely effective in reducing the number of vulnerable individuals. The relative importance of rent and mortgage suspensions, (likewise, widespread COVID-19 policies), in alleviating vulnerability, is highly country dependent.

## Introduction

The COVID-19 outbreak has brought on, alongside a major health crisis, dramatic economic shocks to European countries. Governments around the continent have taken different measures to face the pandemic while preserving jobs and incomes. This paper analyses households’ pre-existing vulnerabilities to an income shock and assesses the degree of protection awarded to employees, in different European countries, by COVID-19 employment protection schemes.

Financial vulnerability has received considerable attention lately, especially after the 2008 crisis. The concept is defined broadly as the likelihood that an economic shock will result in a substantial decline of individual well-being. Risk of or uncertainty about falling into hardship is the key component that differentiates vulnerability from a state of, for example, poverty (Hoddinott and Quisumbing 2010 and Whelan 1993).

To measure financial vulnerability, the literature uses both objective and subjective approaches. Objective measures are independent of the individual’s opinion and may be self-reported or obtained from an external source. Examples include possession of financial assets, amounts in savings accounts, access to credit, or having health insurance. Subjective measures instead are based on perception and self-reported, usually through surveys. An example of the latter is Lusardi et al. (2011) who examine US household financial vulnerability by asking how confident individuals are that they could come up with $2000 in 30 days to face an unexpected need. In this paper, we develop an objective measure. Instead of asking individuals whether they would be able to cope with a hypothetical shock, we assess whether they can cover their usual basic expenditures under a hypothetical shock. In particular, we analyse whether households can afford basic expenditures if deprived of their privately earned income, resorting, instead, to a combination of their savings and publicly provided income such as pensions and public transfers.^1^ We firstly consider only food and utilities as basic expenditures and then extend this basket to include mortgages and rents on main residences, for individuals who own no other residence^2^.

We build on Midoes (2020), which provides an estimate of the number of households that could not afford basic expenses without privately earned income. Here we simulate net incomes obtained under the actual COVID-19 unemployment protection schemes enacted by each country, and assess whether individuals can afford basic expenditures.

We analyse 7 countries: Austria, Belgium, Finland, France, Germany, Italy and Portugal. We calculate financial vulnerability using Wave 3 of the ECB Household Finance and Consumption survey (HFCS), conducted in 2017.

Taking a three months horizon, which is the average length of the lockdown measures enacted in the countries under analysis during the first COVID-19 wave, 31.2 million, or 13% of the population of the countries considered, would not be able to cover those expenses. When we add rent and mortgages on the main residence, almost 20% become vulnerable at the 3 month mark. Results for one, six and twelve months are included in the Appendix.

Our results highlight the importance of unemployment benefits: 5.9% of individuals in households that would be affected by a loss of employee income, without support, are unable to cope with food and utilities in a three months horizon. Once all COVID-19 unemployment benefits enacted by the different countries are considered, only 1.1% of individuals possibly affected would not be able to cover for these expenses. By expanding the basket to include rent and mortgages on the main residence, the numbers are respectively 10.5%, and 2.1% once the COVID-19 unemployment benefits are awarded.

This vulnerability characterization is useful for economic research and public policy, being simple and readily available from survey data covering consumption and savings. It can help identify groups of individuals more vulnerable to income shocks, distinguishing, for instance, between employees and the self-employed. It can also be used to describe by how much households will have to reduce savings to keep basic consumption constant. Moreover, the measure of vulnerability is statistically positively associated with being an immigrant and a single parent, amongst other risk factors, routinely found in the literature to be associated with other measures of financial vulnerability. This shows it can be indeed a useful indicator.

The rest of the article is structured as follows. In Sect. 2 we summarise the literature on financial vulnerability and the rationale for our specific measure. Section 3 describes the data and methodology, giving details on our financial vulnerability measure. Section 4 presents results, firstly describing the vulnerability of the population to a shock to privately earned income, and then how it differs amongst sociodemographic groups. We then consider the vulnerability of the population to a possible shock to employee income, and how much COVID-19 employment protection schemes, the largest support measure, attenuate vulnerability (and how much more they could attenuate it in each country, if the benefits were more generous than those in place). Finally, we consider the decrease in vulnerability associated with mortgage and rent suspensions, likewise, widespread COVID-19 support policies. Section 5 concludes.

## Related Literature

Financially vulnerable households are those particularly exposed to shocks, such as job loss or reduction in working hours, that can eliminate or reduce an income source (Anderloni et al. 2012). This condition is associated with negative mental and physical health status (both self-rated health and as judged by clinicians) (De Witte et al. 2015), with multiple chronic conditions such as hypertension, heart disease and depression, and with decreases in overall subjective well-being (Sjöberg 2010 and Ferrie et al. 2001) and even with poor family and marital functioning (Larson et al. 1994). The impact of the COVID-19 pandemic and the economic crisis has been unequally distributed. Women, the young, and those with lower levels of education are bearing the brunt of the downturn in terms of job and earnings losses (Adams-Prassl et al. 2020a and Adams-Prassl et al. 2020b ). In this context, Codagnone et al. (2020) highlight how financial vulnerability increases mental distress in different European countries and Simha et al. (2020) finds a significant influence of gender, with vulnerable women experiencing significantly greater distress than men.

The measure of vulnerability used in this paper is based on the idea that when deprived of their labour and capital income, individuals resort to a combination of savings and remaining sources of income (such as publicly provided income or capital income) to cover for basic expenses. Yet this is not the only way people can face an economic shock. Arguably, they can adapt their consumption patterns, by, for example, reducing their demand for certain types of goods (Hamermesh 1982) or increasing their home production of goods (Aguiar and Hurst 2007).

Even though individuals might adapt their consumption when faced with a financial shock, their ability to do so is constrained. Many households have “consumption commitments”, for instance, in what pertains to housing, that are costly to adjust, especially in the short-term. Indeed, most homeowners do not move during unemployment spells and have mortgage or rent payment commitments (Chetty & Szeidl 2007). Consumption reacts in heterogenous ways to an unemployment spell. Dynarski and Sheffrin (1987) and Stephens (2001) argue that blue collar workers have shorter unemployment spells and their consumption is less sensitive to a change in employment status. Similarly, other factors such as age, gender and educational level are being linked to differing individual consumption behaviours during the pandemic (Minguez et al. 2020 and Piyapromdee and Spittal (2020)). In Sect. 4 we assess how individual characteristics such as gender, age, educational level, migration status, and percentage/number of non-dependent adults receiving income, is associated with our vulnerability measure. Such analysis can help put into perspective differences in consumption reactions between groups found in the literature.

Another reason why changes to consumption might not come about immediately, is that individuals have consumption habits. Models of habit formation state that the instantaneous utility function of individual *i*, having habit forming preferences $u(c_{i}, x_{i})$, depends not only on current consumption $c_{i}$, but also on the habit level $x_{i}$. In particular, this type of model assumes that only the component of consumption over and above the habit level, i.e., $c_{i} - x_{i}$, contributes to utility (Naik and Moore 1996). Hence, any change in consumption from the habit level is perceived as a gain or a loss (Günther and Maier 2014).

The housing expenses we consider are costly to adjust and so are basic utilities. These expenses, together with food - excluding restaurants -, might be somewhat adjusted, given intertemporal substitution of consumption, yet the elasticity of substitution is expected to be below one. Thus, individuals whose savings are insufficient to keep past consumption levels are vulnerable in the sense that they are more likely to experience larger decreases in welfare.^3^

While certain papers (Muellbauer 2020 and Clayes et al. 2021) have argued that the COVID-19 crisis shock implied a consumption elasticity to income reduction larger than one, these comments, about aggregate consumption, are simultaneously affected by the supply side shock (namely by forced closures or capacity limits). Moreover, those considerations include consumption of non-essential expenses, unlike our basket, for which supply was unaffected.

To meet ordinary living expenses under income shocks, households might also rely on resources other than bank savings, pensions and public transfers - the ones specifically considered in our indicator in Sect. 4.1-, namely, they might resort to consumer credit. This, however, comes at a cost (interest), and can in itself be taken as a measure of financial vulnerability. And even if some families do take out loans to afford housing expenses (Andersen et al. 2020), Horvath et al. (2020) shows that in the United States, since the onset of COVID-19, new supply of credit to risky borrowers is limited. In the presence of liquidity constrains, another important resource to consider are loans or gifts from family and friends, which can, likewise, ensure minimum levels of consumption. While these loans are typically short-term and small (Long 2020), they do provide an additional buffer. Such formal and informal credit could be acknowledged explicitly in future research to better assess short-term financial vulnerability. In Sect. 4.2, when only salaries are affected, individuals still accrue income from any regular transfers from family, just not from ad hoc transfers.

## Data and Methodology

Our analysis uses the ECB Household Finance and Consumption Survey (HFCS), a cross-sectional national survey combining information on income, wealth, liabilities and consumption. We use Wave 3, the most recent wave, released in March 2020 and carried out in 2017. Weights provided ensure the number of households matches the total number of households in each country. The survey provides information on all individuals within each household sampled. Income and expenses have been uprated from their survey reference period to price levels of 2020 through the countries’ consumer price index, such that 2020 policies are applied to simulate disposable incomes. Table 1 in the annex presents summary statistics of our sample.

We present results for 7 countries: Austria, Belgium, Finland, France, Germany, Italy and Portugal. These countries exhibit different starting situations. The COVID-19 unemployment benefits enacted differ considerably in their generosity amongst the countries, which can be partly attributed to the different fiscal space available; financial security of households is also substantially different amongst the countries. This allows us to have a picture of how different country-specific elements affect vulnerability to the COVID-19 shock.

Given our data was collected in 2017, we followed Christl et al. (2020) to model through EUROMOD labour market transitions from employed to unemployed and vice-versa. This simulation was made on the basis of aggregate statistics using (informed) random allocation. These results are similar to the ones obtained without labour market transitions.

Given HFCS data only covers gross income, in Sect. 4.2, we derive net incomes through the EUROMOD microsimulation model. EUROMOD is a static model that calculates country-specific social insurance contributions, income taxation and means-tested cash benefits to obtain market incomes. It simulates cash benefit entitlements and direct tax and social insurance contribution liabilities, on the basis of the tax-benefit rules in place and information available in the underlying datasets (see Sutherland (2001)). We build on Boone et al. (2019) and Kuypers et al. (2017) to convert HFCS data into a EUROMOD database to obtain disposable income. With a few exceptions, the majority of the variables needed for the simulations of taxes and benefits are available in the HFCS data. When the simulation of a contribution or a benefit was not possible to simulate through EUROMOD it was omitted. For example, HFCS does not include information about the firm size for the calculation of employer contributions neither cadastral income of the main residence for the calculation of property taxes. Boone et al. (2019) point out that those benefits are small and received by a limited number of people with a small impact on the overall household disposable income. Nonetheless, EUROMOD based on the HFCS slightly “undersimulates” taxes and social contributions paid by households (Kuypers et al. 2016) and hence might slightly overestimate household disposable income.

The HFCS data consists of 5 implicates, with small differences between them. Following the HFCS methodology, our point estimates are the averages across the 5 implicates. Confidence intervals are calculated based on between and within imputation variance following the specification proposed in ECB (2020), and either reported in the figures themselves or in Table 8 for readability. Within imputation variance is obtained through bootstrap weights, provided by HFCS, and generated a la Rao et al. (1992). The short regression analysis to better identify risk factors for vulnerability, likewise, uses between and within imputation variance through bootstrap weights and multiple imputation.

We determine, in each implicate, which households in the sample cannot afford their typical expenses with certain types of resources (e.g., only with their bank deposits), as explained below, and count the number of individuals living in such households. We then extrapolate to the countries’ population by weighing each individual within a household by that household’s weight. We report the average across the five implicates of the weighted number of individuals living in households which cannot afford expenses.^4^

Although, from a life-cycle perspective, it is not possible to be perpetually vulnerable with this measure of vulnerability, this is not necessarily an issue. As mentioned in the literature review, in the presence of a shock, households have for example commitments and habits that prevent them from immediately changing their consumption. But in a life cycle perspective, once time is included, households can change their basket of expenses into one that is affordable in the new scenario. Furthermore, the measure proposed here is meant to assess the likelihood of a decline in individuals’ well-being in the presence of a financial shock. If, in the middle term, the household adapts its basket of goods by reducing expenses, it will indeed move to a lower indifference curve and suffer a reduction in welfare. Once the transition is complete, the reference point of this household to evaluate the impact of future shocks will be this new indifference curve. Of course, in the middle term, also the income shock of COVID-19 might be attenuated, either through different household financial decisions or macroeconomic improvement. The status of financial vulnerability, as we construct it, is thus not static.

### Determining Whether Households can Afford Expenses

To determine whether a household is financially vulnerable, we divide the resources a household has available in *m* months (pooled resources of all household members) by a basket of expenses in *m* months (pooled expenses of all household members). If the ratio is below one, the household cannot afford these minimal expenses in the short run (without adding external resources such as debt or ad hoc help from family members, which is not necessarily readily available and/or creates future financial commitments).

We thus construct a dummy variable $vulnerable_{h}^{m}$ which, for each household *h* in an *m*-month time horizon, equals one if the ratio is below one (meaning that the household pooled expenses are higher than the household pooled resources), and zero otherwise:

1 $$\begin{aligned} vulnerable_{h}^{m} =\left\{ \begin{array}{ll} 1, &{} \quad \text {if}\ ratio_{h}^{m} = \frac{pooled\_resources_{h}(m)}{pooled\_expenses_{h}(m)} < 1 \\ 0, &{} \quad \text {otherwise} \end{array}\right. \end{aligned}$$

We will work with several versions of the denominator ${pooled\_expenses_{h}(m)}$. A first version aims to capture vulnerability by considering the most basic expenses: those with food at home and with utilities (comprising electricity, water, gas, telephone, internet, and television). A second version includes rents and mortgages on the household main residence. For the latter, we consider loans contracted to purchase, construct, refurbish or renovate the household’s main residence. We change the denominator in this way only for households which own no other residential properties. The objective is to capture only the most vulnerable individuals, who would not have an alternative residence in case they were not able to face housing expenses. Some of the individuals we exclude might also have no alternative residence if their properties are for instance rented out, so the exercise is conservative. A scenario where properties beyond the main residence are considered is also provided.

The numerator ${pooled\_resources_{h}(m)}$ always considers household bank deposits. We change it in an additive way, adding different household resources, while keeping the denominator fixed, and thus identify fewer and fewer households not able to afford expenses. In Sect. 4.1, as household resources, we consider (beyond bank deposits) public pensions, unemployment benefits and other (pre-COVID-19) public transfers. Separately, in Sect. 4.2, we determine whether households can afford expenses when individuals are deprived of salary income and when they are deprived of salary income but awarded COVID-19 employment protection income. In this case, all other income, such as capital income and regular private transfers, are not reduced. We do this exercise to all households where at least one individual accrues employee income. As data on characteristics of those affected is released and analysed, extensions could simulate a shock which more closely reflects the actual COVID-19 shock, which did not affect all employees equally. This could be done by, for instance, considering disaggregation by sector of employment to allow for different probabilities of actually facing reduced income. Our exercise, differently, is a generalization, of pre-existing vulnerabilities to an unexpected shock, and of the potential limitations of the developed government support measures. The buffer provided by sales of liquid assets (beyond deposits) is considered in Sect. 4.4.

Under our definition, only very few households are vulnerable before any shock to their income, i.e., very few are unable to cover for basic expenses with their current reported disposable income^5^$$^{,}$$^6^. Thus, we report results in percentage of the population, without subtracting these small baseline percentages.

Our main results take as reference the three months horizon, which is the average length of the lockdown measures enacted in the countries under analysis during the first COVID-19 wave.^7^ In addition, three months of income or expenses is the minimum that many planners recommend individuals hold as emergency funds (see Lusardi et al. 2011 and Collins et al. 2015). Results for 1 month, 6 and 12 months are presented in the Appendix.

Precautionary savings in cash are another resource not explicitly considered. Our analysis overall considers only households which have a bank account, as these households are less likely to use cash as an important source of savings (Kendall 2010). As a sensitivity analysis, we kept only households which have a bank account, but allocated cash to individuals above 18, according to the percentage reported in Esselink and Hernández (2017) as having precautionary cash savings in the country. We added such cash savings to their household available savings. In all the EU countries covered, less than than 40% of the population keeps cash as precautionary savings outside of a bank account. In most countries, the percentage is below 36%. Furthermore, according to Esselink and Hernández (2017), those who do keep cash outside of bank accounts keep relatively low amounts: 23% state having less than 100€, 22% between 100€ and 250€ and 19% between 250€ and 500€. Our estimates, as a result, remained very similar, and are not shown. Yet, it should be noted that such question is necessarily sensitive, with individuals more likely to omit and underestimate cash holdings.^8^

### COVID-19 Unemployment Benefits

In most of the countries under analysis, the COVID-19 unemployment benefits are defined as a percentage of the employee’s gross salary, with a floor and a ceiling. In the case of Austria, the benefit includes a public net income replacement of between 80% and 90%, while in Germany, it varies between 60% and 67%, according to the region. In Finland, the benefit is a daily fixed amount, which can be increased according to the number of children in the household. Table 2 in the Appendix summarises the main characteristics of the COVID-19 unemployment benefits simulated for each country. We decide to look at the effect of these benefits in safeguarding employees against vulnerability, because this support, which is essentially a more generous form of the usual unemployment benefits, is the largest COVID-19 support policy in all countries. Our sample in this part of the analysis is reduced to households where someone accrues employee income.

Besides applying income taxation, in the scenario where individuals are not receiving salaries, they also receive means-tested social benefits to which they would be entitled under reduced income (as simulated by EUROMOD). We awarded COVID-19 benefits to all employees accruing a yearly salary income of at least twelve times the minimum wage, as they are more likely to have been in full employment the previous year and thus entitled to coverage. Although some countries require less than a year as employees to award COVID-19 unemployment benefits, we adopted this criteria of eligibility given that individuals are asked about their gross income over the past twelve months.

For an affected household, i.e., where someone accrues salary income, household net income differs between the scenario with no salaries and the scenario with no salaries but with COVID-19 employment protection benefits because of two types of individuals: i) individuals who, instead of accruing their normal employee income above the minimum wage, now receive a part of it as defined in the COVID-19 policies and ii) individuals who reported an employee income below the minimum wage, who now receive a zero employee income under our simulations.

## Results

### Vulnerability Without Unemployment Protection Schemes

Considering the seven countries of our sample, Austria, Belgium, Finland, France, Germany, Italy and Portugal, we estimate that 31.2 million of individuals–or 12.8% of their population - are financially vulnerable when we consider food and utilities, meaning that they would not be able to afford those expenses for three months without privately earned income.

**Fig. 1:**
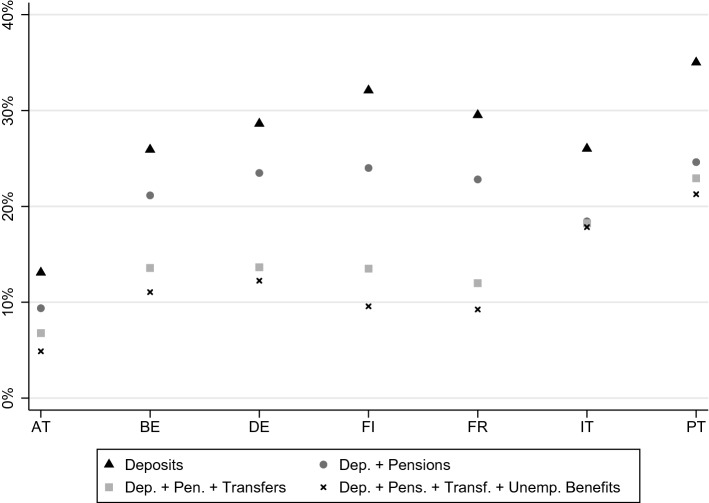
Percentage of vulnerable individuals in a three months horizon, considering food and utilities, resorting to deposits and different sources of income

In Fig. 1, we plot, for each country, the percentage of vulnerable individuals, and how the availability of resources beyond deposits decreases vulnerability. There are stark country differences. In Portugal and Italy, after considering deposits, pensions and public transfers, 21.5% and 17.8% of the population could not afford food and utilities if they were deprived of their privately earned income. This is more than double the proportion of vulnerable population in Austria. The role played by public transfers is also very heterogeneous across countries: while in Finland they seem crucial, reducing the percentage of vulnerable individuals by more than 8 percentage points, in Italy they play almost no role as a buffer for families which currently receive them.

**Fig. 2:**
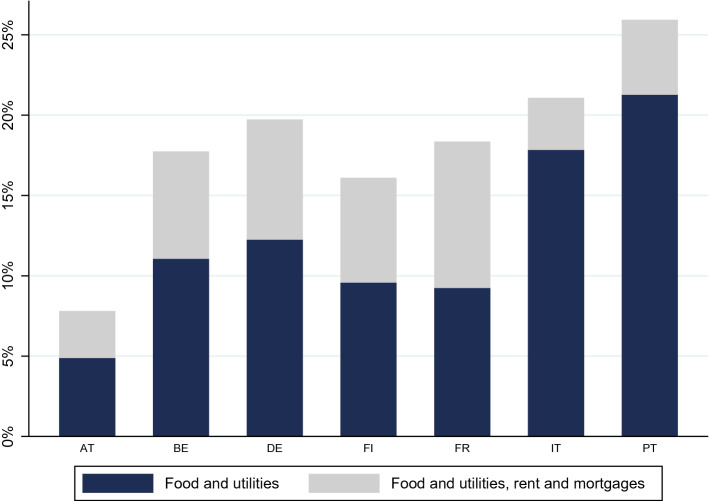
Percentage of vulnerable individuals in a three months horizon when rent and mortgages are added to food and utilities

Both rent and mortgages expenses substantially increase the number of vulnerable individuals: considering deposits, pensions and public transfers, 47.2 million, or 19.4% of the population analysed, are vulnerable when we add rents and mortgages to the basket of basic expenses (see Fig. 2). The impact of housing expenses is more severe in some countries such as Germany and France. As highlighted in Midoes (2020) and discussed in more detail in Sect. 4.3, this suggests that rent and mortgage suspension can be an effective policy to alleviate household vulnerability in some countries.

We focus on the percentage of vulnerable individuals to give an estimate of the scale of vulnerability in a country, and of how encompassing developed income support should be. The ratio of resources to expenditures itself - as opposed to our main measure of choice, a count of whether such ratio is below one - is very unequally distributed within a country, and thus, while summary statistics on these ratios are informative for the overall resilience of the population, they do not capture the most vulnerable specifically. Even so, the mean ratios still illustrate effectively differences across countries. For example, taking deposits, pensions, public transfers and normal unemployment benefits as household resources, and food, utilities and housing expenses as expenditures, Italy exhibits at the three month markt the lowest mean ratio of resources to expenditures, of 7:1, while the mean ratio is highest in Austria, at 15:1 (see Table 3 for further details).

The likelihood of being vulnerable differs substantially across sociodemographic groups. Table 4 shows how certain groups are more at risk than others - of note, immigrants. Yet, since several of the divisions of interest considerably overlap (say, number of children, migration status, age of adults in the household), we use a linear regression to determine which variables are associated with vulnerability status beyond their correlation with other potential risk factors (a linear probability model). Results are shown in Table 5.

Individuals who were born outside the country where they reside, and particularly those born outside of the EU, are approximately 9 p.p. more likely to be vulnerable.

Households where adult individuals are older are less likely to be vulnerable, which is to be expected given capital accumulation and income levels increase with experience and age. Each additional year in the average age of non-dependent adults is associated with a decrease of between 0.4 and 0.5 p.p. in the likelihood of being vulnerable. Having two potential earners as opposed to only one, in the case of working-age adults, is also associated with decreased vulnerability, as expressed by how a household of two working age adults without children is 2.8 p.p. less likely to be vulnerable than a household of only one. Having two, not potential, but actual, earners (individuals who made some income from labour or pensions in the last year), as opposed to one, is associated with a 6 p.p. lower probability of vulnerability^9^.

Single parent households are 4 p.p. more likely than single young-age adult households to be vulnerable. Even when already considering the typology of the household, being a man is still associated with lower probability of being vulnerable.

### Vulnerability Under COVID-19 Unemployment Benefits

In this section we consider whether households can afford expenses when they are deprived of salary income (only of salary income, instead of all privately earned income as in the preceding section) and when deprived of salary income but awarded COVID-19 unemployment support measures as enacted in each country. We restrict our sample to households where at least one individual has been in employment throughout the previous year. It should be noted then that percentual results are in percentage of individuals living in such households. We first assess vulnerability through individuals in households unable to afford basic expenses without salaries. We compare this scenario to one in which individuals are receiving COVID-19 unemployment benefits. In both cases, our final income metric is net income, obtained through the microsimulation model EUROMOD.

**Fig. 3:**
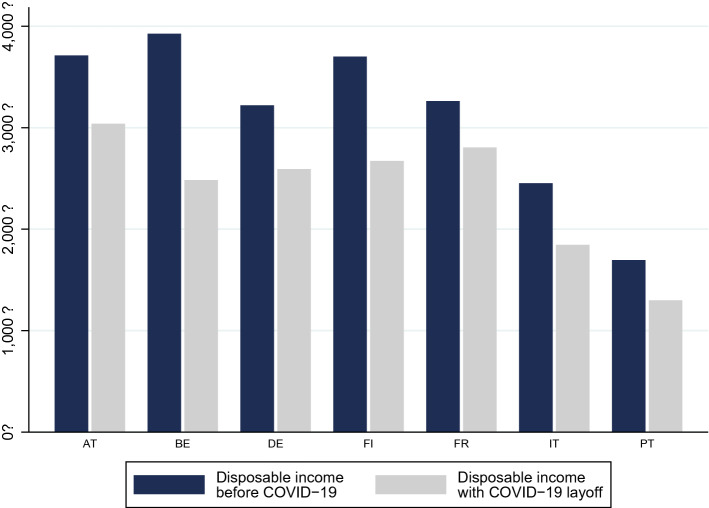
Average household disposable income for households earning salary income, before COVID-19, and with COVID-19 layoff but no other salary income (in €)

How net income, on average, is reduced, can be seen in Fig. 3, where we present mean disposable household income before COVID-19 layoff (that means, assuming individuals receive their normal salary) and after we eliminate their usual salary income but award them COVID-19 benefits, for households affected (those who receive employment income). While in countries like France and Portugal, average incomes with COVID-19 layoff are only 25% lower, Belgium stands out, with average household income under the COVID-19 layoff scenario being less than half the status quo average household income.

**Fig. 4:**
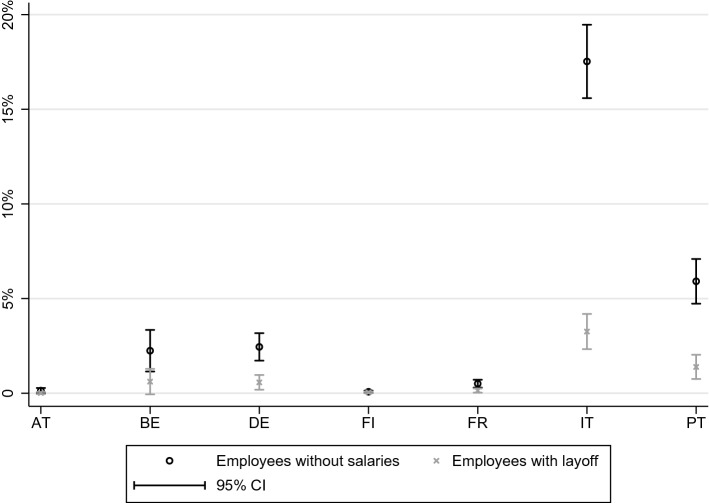
Percentage of vulnerable individuals with and without COVID-19 layoff, in a three months horizon, considering food and utilities

**Fig. 5:**
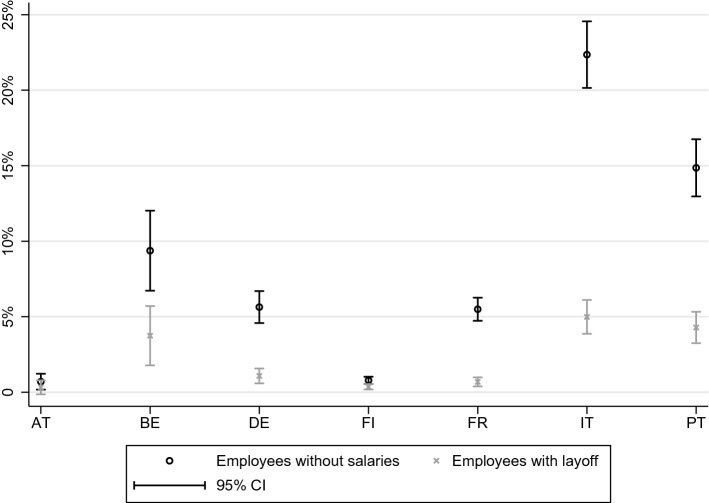
Percentage of vulnerable individuals with and without COVID-19 layoff, in a three months horizon, considering food and utilities and rent and mortgages on the main residence

We find that, considering food and utilities only, the situation is not too dire overall: only 5.5% of affected individuals are vulnerable at this point. This number hides important country differences, specifically, that in Italy, 17.5% of affected individuals are vulnerable. Figure 4 shows the percentage of vulnerable individuals when salaries are removed and when COVID-19 unemployment benefits are subsequently added, by country.^10^ For all countries the situation improves substantially. In Italy, there is a drastic reduction to only 3.3% of vulnerable individuals. For all the other countries, less than 1.5% remain vulnerable. While we estimate that 9.0 million people in the countries considered could not afford food and utilities, only 1.8 million would not be able to once COVID-19 unemployment benefits are awarded.

Even if in Italy only 3.3% of the individuals considered are vulnerable under the COVID-19 layoff scenario, it is the country with the highest absolute number of vulnerable individuals: 1.2 million. Germany, where only 0.6% of those affected are vulnerable, nonetheless follows, with 341 thousand. These vulnerabilities result both from a lack of savings and from reduced income directly.

When we consider rents and mortgages as part of basic expenses, the percentage of vulnerable individuals increases to 9.8%. Nonetheless, the COVID-19 employment protection benefits reduce the percentage of vulnerable individuals to 2.1%. As Fig. 5 shows, the benefits are again quite effective at reducing vulnerabilities across all countries. In Italy, Portugal and Belgium, where vulnerability is highest at baseline, we estimate only 5.0%, 4.3% and 3.7% respectively of those affected are vulnerable at the three month mark.

In our simulations, we considered that employees who earn less than the minimum wage are not entitled to the employment protection benefits. We find that looser employment contracts, besides connected to lower possibility to access the benefits as designed by countries, are also associated with higher vulnerability under our measure. Individuals with temporary contracts receiving below the minimum wage, are up to twice as likely to not be able to face food, utilities and essential housing expenses if deprived of salaries (see Table 7).

#### How Much Would Higher Unemployment Benefits Help?

With the exception of Finland, - where the unemployment benefit is a daily fixed amount-, the unemployment scheme covers a percentage of salaries, varying between 60% (in Portugal) and 90% (for certain subgroups in Austria). By allowing for a higher income replacement rate than under usual unemployment benefits, countries try to protect jobs and mitigate the economic shock by sustaining consumption levels (Schnetzer et al. 2020). While the percentage of vulnerable individuals is already low when considering country-specific coverage rates, higher rates of coverage, at 90% of salaries, would, logically, substantially decrease vulnerability.

Figure 7 reveals how affected households would be much less vulnerable under such, more generous, unemployment benefits. The increase has a more noticeable effect in Italy, Portugal and Belgium, where the percentage of vulnerable was still above 3.5% with current rates. Nonetheless, there are also meaningful reductions in countries with an already extremely low percentage of vulnerable individuals, such as France and Finland.

In our microsimulation exercises, individuals were vulnerable both because the reduced monthly income is not enough for basic expenditures, and because of low savings. For most households, their monthly income becomes insufficient to cover for expenses under the layoff scheme not because they were deemed ineligible to receive it under our exercise, but because the proportion of income ensured, together with savings, is insufficient. In Portugal and Italy, 62% and 58% of individuals vulnerable at the 3-month mark are in households where all individuals were either deemed eligible for the COVID-19 support (accruing more than the minimum wage) or unaffected (because they did not accrue any employee income). In France, 80% of vulnerable individuals are in such households. In Belgium, Germany and Austria, all individuals deemed vulnerable are in this situation. The vulnerable individuals identified in our simulations do not come from gaps in coverage generated by our procedure, but from the coverage itself not being sufficient for expenses. The results we now find, where there is an important reduction of vulnerability under a more generous layoff scheme, are a reflection of this. The results showed Belgium with a percentage of vulnerable individuals on par with Finland and France and slightly behind Austria, when we considered deposits, pensions and pre-existing public transfers. However, once the COVID-19 layoff is considered, Belgium only performs (slightly) better than Italy and Portugal. This is because the Belgian COVID-19 income support is amongst the least generous (as detailed in Fig. 3 on the previous section).

### Vulnerability Under Mortgage and Rent Suspension

**Fig. 6:**
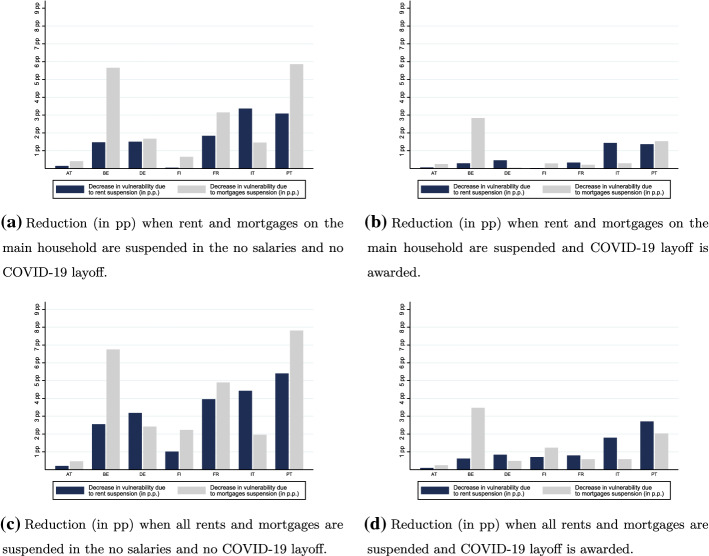
Effect of mortgages and rent suspension

Rents and mortgages suspensions have been two widely discussed and applied policies in several European countries. The conditions necessary for a mortgage or rent suspension vary amongst countries. The upper charts of Fig. 6 shows how vulnerability is reduced (in percentage points-pp) when we consider only rent and mortgages on the primary residence, and the bottom charts, when all rents and mortgages are considered.

In a scenario where employees are not receiving their salary, suspending rents and mortgages on main residences would reduce the number of vulnerable people by 7.1 million. With the exception of Austria, (where vulnerability a priori is much lower), rent and mortgages suspension are an effective policy, even when only the primary residence is eligible (a suspension of rents and mortgages beyond these does not meaningfully alleviate vulnerability, as can be seen by the small differences between the upper and lower panels). Rent suspension is only more effective than mortgage suspension in Italy; in Belgium, France and Portugal mortgages suspension is more effective instead. Awarding individuals with the COVID-19 unemployment benefit reduces the percentage of vulnerable individuals in all countries and, with the exception of Belgium, makes the difference between mortgage and rent suspension, in terms of vulnerability alleviation, slimmer.

### Liquid Assets as a Buffer

To face expenses in the short-run, households might resort not only to deposits but also to other liquid assets, which can be easily sold. Besides deposits, households can resort to their mutual funds, bonds, non self-employment private businesses (though their liquidity is more conditional on economic circumstances), shares and managed accounts. Financial income from these investments, which they earn regularly, were left unaffected by the COVID-19 related simulations from section onwards.

The prevalence of these assets across EU countries, though quite variable, is generally low, particularly for lower income and lower wealth individuals. Since we are focusing on individuals who, without deposits, would not be able to cover for the most basic expenses for three months, we are analysing a quite low deposit wealth group, but a low-wealth group generally. In its majority, the group considered holds assets only in the form of deposits and on occasion real estate, a quite illiquid asset. The possibility to earn income from real estate while remaining in the same residence, through instruments such as reverse mortgages, is underdeveloped in the countries considered.

Thus, when we consider sale of liquid assets beyond deposits as a resource to cover expenses, the percentage of vulnerable individuals decreases only very slightly. In certain countries where these other liquid assets are more prevalent, specifically in Finland, the percentage of individuals unable to cover for 3 months of expenses with liquid assets is 8 p.p lower than when resorting to deposits alone. If Finnish households have available deposits, pensions and public transfers, adding other liquid assets reduces the vulnerable by only 0.7 p.p. Indeed, once we consider households might use not only deposits or all liquid assets, but also pensions and public transfers, using all liquid assets instead of other deposits leads to only residual reduction in the number of vulnerable individuals. After Finland, the reduction ranges from 0.1pp in Austria and Portugal to 0.5pp in Germany.

**Fig. 7:**
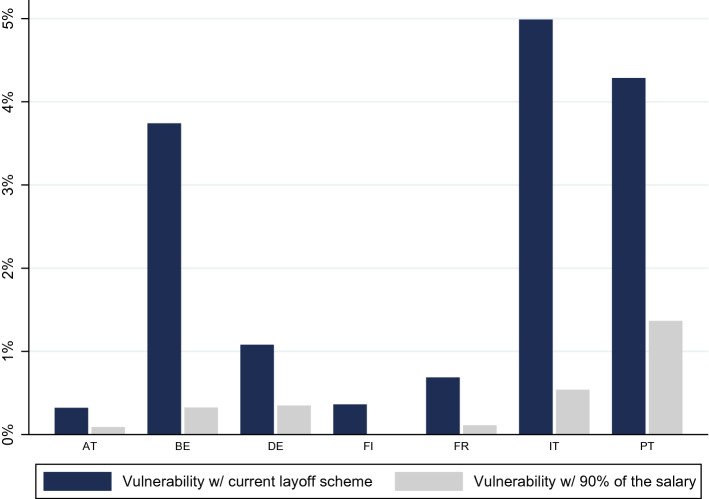
Percentage of vulnerable individuals with the current COVID-19 layoff and with a replacement rate of 90% of salaries, in a three months horizon, considering food and utilities and housing expenses

## Discussion and Concluding Remarks

This paper suggests an indicator of financial vulnerability, readily available from survey data, and how to use it to provide evidence on the sensitivity to an income shock such as that inflicted by COVID-19, and on how effective the enacted government support policies might be.

As per the indicator, certain groups are especially likely to be financially vulnerable-immigrants, particularly those born outside of the EU; single parents; single-earner households; less educated households; and women. These associations show typically vulnerable groups, vouching for the robustness of the indicator. Certain groups such as immigrants might also be more exposed to job losses under macroeconomic shocks and have increased difficulty in accessing extraordinary government support, making specifically designed policies advisable.

Public pensions, a source of income largely unaffected by COVID-19, are essential to cover basic expenses for many of the households who receive them. The effect of other public transfers is more heterogeneous. They have little effect beyond pensions in some countries, namely Portugal and Italy, but play an important role in France, Belgium and Germany. Even with pensions and public transfers, a large number of individuals depend on household privately earned income to cover for their most basic expenses in a short term: 47.1 million individuals, or 29%, of the population of the seven European countries analysed, cannot cover for three month of food, utilities, rent and mortgages by resorting to their deposits, pensions and public transfers.

The largest COVID-19 support policy has been the employment protection schemes, essentially, more generous versions of the usual unemployment benefits. By restricting our sample to households where someone accrues employee income, we assess how effective these measures might be in alleviating vulnerability of this subgroup. In all countries, as expected, we observe a very large drop in the number of vulnerable affected population when we award them COVID-19 unemployment benefits. When we consider net incomes and the dependence on employee income specifically, we find significant differences across countries. Employees in Austria, Finland and France are less vulnerable to a labour income shock than employees in Italy and Portugal. The employment protection schemes awarded are extremely effective in decreasing these numbers, particularly in Italy. Indeed, considering food and utilities, rent and mortgages, only 5.0% of those affected remain vulnerable at the 3 month mark when receiving the designed subsidies, implying a reduction of almost 17 percentage points. A sizeable reduction is also observed in Portugal where the vulnerable population decreases from 14.5% to 4.3%.

There are two reasons for the greater effectiveness of the schemes in countries like Austria and Finland than in, for example, Italy. The first is that, in the former two countries, only 0.3% receive, under the scheme, an income below basic expenses, while in Italy, 13.1% do. The difference in the generosity of the support results in a more effective public transfer in the former two countries, just as with non-COVID-19 public transfers. The second reason is the pre-existing differential in savings - Finish and particularly Austrian households can cover for their expenses from deposits alone for a longer period of time.

An important reduction in the percentage of vulnerable individuals can be achieved by providing a more generous layoff scheme. Considering only food and utilities, and setting a rate of coverage of 90% of salaries, implies 187 thousands of individuals falling into vulnerability, which represents around 0.1% of the total population analysed. This number is considerably smaller than the 1.7 million individuals that could fall into vulnerability under the current layoffs schemes of each country.

Rent and mortgage suspensions are more effective in some countries than in others. Countries like Portugal, Belgium and France can achieve an important reduction household vulnerability through these measures, while in Italy, they should be coupled with COVID-19 unemployment benefits to bring about a meaningful reduction. If Italy applies the COVID-19 layoff, it would have the same percentage of vulnerable individuals as Portugal when just rent and mortgages suspension are applied.

Importantly, even when considering the suspension of all loan repayments in tandem with the layoff, there is still a larger percentage of individuals in Italy and Portugal unable to keep the remainder of their usual monthly expenses, than in the rest of the countries without any such measure. Taken jointly, the differential between countries remains. When analysing vulnerability under income support and suspensions, we should bear in mind that these measures are not analogous. A loan repayment suspension is only a suspension, entailing payments in the future. In the short-term, it might serve the same purpose as income support-reducing vulnerability-yet, since it entails repayment in the future, it is comparatively less effective in fueling future consumption, a possible public policy goal.

Countries with wider fiscal space can enact more generous policies. The layoff schemes in Austria or Finland are more generous than the Portuguese. Households in these countries, per our results, are the most robust to overall income shocks, being able to sustain expenses from deposits alone for longest. As a result of differing fiscal space, rather counter intuitively, it is in the countries where individuals least require income support for basic needs that states make available substantially more generous subsidies. These differentials in suffered impact, household finance, and public finance, underlie the different perceptions of urgency about the crisis and different eagerness for concerted EU-financing of support policies.

**Table 1:** Summary statistics

	AT	BE	DE	FI	FR	IT	PT
Median monthly gross income (in €)	3436.2	3625.2	3345.0	3393.7	2691.7	2083.4	1463.5
Median total deposits (in €)	12,724	10,857	7700	5000	6709	5000	3000
Median deposits in sight accounts (in €)	1276.2	1641.0	1800.0	3000.0	1000.0	1072.0	1064.0
Median deposits in savings accounts (in €)	13,380.8	15,185.4	10,000.0	10,000.0	7000.0	10,000.0	10,000.0
Deposits/(food + utilities) (mean)	45.7	53.2	41.3	42.0	34.0	20.8	34.8
Deposits/(food + utl. + rent + mort.) (mean)	38.1	45.2	33.1	34.4	26.9	19.6	32.0
Deposits/(gross income) (mean)	9.2	11.2	14.5	7.6	12.4	14.8	14.7
Disposable Income (per capita mean in €)	3712.8	3927.4	3220.4	3701.9	3262.9	2452.7	1695.8
Disposable income without salaries (per capita mean in €)	2300.4	1788.1	1294.7	2511.5	1949.5	1134.9	756.8
Disposable income without salaries but with COVID-19 layoff (per capita mean in €)	3039.1	2484.1	2592.0	2673.0	2805.1	1845.2	1298.7
Sample-individuals in households with employees	4544	3346	7938	18,923	23,052	10,915	10,689
Sample-households	3072	2329	4942	10,210	13,685	8156	5924
Sample-individuals	6414	5370	11,251	24,818	32,799	19,366	15,079
Weighted sample-individuals in households with employees	5,940,698	7,695,731	59,400,344	3,758,227	38,470,335	42,439,412	7,460,374
Weighted sample-households	3,922,652	4,773,882	40,086,024	2,677,099	29,217,223	23,741,518	3,971,316
Weighted sample-individuals	8,402,665	11,320,683	81,693,496	5,418,579	60,782,668	65,526,523	10,309,573

Results computed at the household level except for disposable income, which is calculated at the individual level, taking as sample the individuals in households with employees

**Table 2:** Details on the COVID-19 unemployment benefits simulated Sources: Andersen et al. (2020) and European Commission (2020)

	Fiscal response
Austria	Percentage of salary coverage ranges from 80% to 90% of previous net monthly earnings, according to their level. If gross monthly earnings of the previous month were above 5,370 €, there is no public compensation
Belgium	Percentage of salary coverage is 70% of gross salaries, with a minimum of 1,591.72 € (national minimum wage) and a maximum of 2,074.80 €, and subject to income taxation of 15%. An additional 5.32 € per day are awarded to individuals
Finland	33.66 € per day, gross, are awarded to individuals, plus an additional daily subsidy of up to 10 €, according to the number of children in the household
France	Percentage of salary coverage is 70% of gross salaries, with a minimum of 1219 € (national minimum wage) and a maximum of 5485.5 €
Germany	Percentage of salary coverage is, during the first four months, 60% of net income, or 67%, if there are children in the household. For benefit calculation, monthly gross wages are capped at 6,900 €. Between the fourth and seventh month it increases to 70% and after the seventh month to 80%
Italy	Percentage of coverage is 80% of gross salaries. If salary is below 2,159.48 € contribution is capped at 939.89 €; if it is above, contribution is capped at 1,199.72 €
Portugal	Percentage of coverage is 66% of gross salaries with a minimum of 635 € (national minimum wage) and a maximum of 1,905 €

**Table 3:** Mean ratio of resources to expenses, considering deposits, public transfers, pensions and normal unemployment benefits as resources, and food, utilities and housing expenses, for one, three, six and twelve months

	AT	BE	DE	FI	FR	PT	IT
M1	41.2	40.4	31.8	30.3	25.9	30.3	19.3
M3	14.8	14.3	11.3	11.3	9.5	10.7	7.2
M6	8.2	7.8	6.2	6.5	5.4	5.8	4.2
M12	4.9	4.5	3.6	4.1	3.4	3.3	2.6

**Table 4:** Relative risk of vulnerability by sociodemographic group

	Relative risk, born elsewhere in the EU	Relative risk, born outside EU	Relative risk, $\le$ secondary education	Relative risk, 12 years or below	Relative risk if $\le$ 50% of non- dependent adults are earners
AT	2.40	2.43	1.16	1.21	2.72
BE	1.33	2.31	1.18	1.08	2.34
DE	1.92	1.74	1.29	1.36	2.30
FI	1.47	1.43	1.20	1.15	2.46
FR	1.25	1.49	1.19	1.21	3.51
IT	2.08	2.56	1.44	1.46	2.72
PT	0.98	1.60	1.25	1.25	4.29

Vulnerability is measured at the three month mark, resorting to deposits, pensions and public transfers. **“Relative risk, born elsewhere in the EU”** is the ratio between the percentage of vulnerable individuals born elsewhere in the EU and the percentage of vulnerable individuals born in the country. **”Relative risk, born outside the EU”** is the ratio between the percentage of vulnerable individuals born outside the EU and the percentage of vulnerable individuals born in the country. **“Relative risk**, $\le$ **secondary education”** is the ratio between the percentage of vulnerable individuals living in households where the highest educated person has at most secondary education, and the percentage of those vulnerable in households where the most educated has achieved tertiary education. **“Relative risk, 12 years or below”** is the ratio between the percentage of individuals 12 or below who live in vulnerable households and the percentage of those aged 13 or above who do. **“Relative risk if 50% or less of non-depedent adults are earners”** is the ratio between the percentage of vulnerable individuals living in households where up to half of its members above 24 years are receiving labour income or pensions and the percentage of individuals living in households where strictly more than half of its members above 24 are receiving labour income or pensions

**Table 5:** Linear probability model on sociodemographic risk factors for vulnerability

Dependent variable: vulnerability indicator at 3 months. (Deposits + pensions + transfers + unemployment benefits) / (Food + utilities + main housing expenses)		
Born outside the EU	0.0918***	0.0897***
	(5.19)	(4.99)
Born in the EU but not in the country of residence	0.0708***	0.0711***
	(3.39)	(3.41)
Male	$-$0.00987**	$-$0.00790*
	($-$3.02)	($-$2.43)
Tertiary education	$-$0.0280*	$-$0.0171***
	($-$1.98)	($-$3.63)
Twelve years or below	$-$0.00895	
	($$-$$0.57)	
More than 50% of non-dependent adults are earners	$-$0.0137	$-$0.0165
	($-$1.17)	($-$1.41)
Average age of non-dependent adults	$-$0.00497***	$-$0.00406***
	($-$24.80)	($-$10.60)
HH of 2 adults above 65 years		$-$0.0482***
		($$-$$3.45)
HH of 3 adults		$-$0.0236
		($$-$$1.60)
HH of 1 adult and children		0.0702**
		(3.18)
HH of 2 adults and 1 child		0.00493
		(0.30)
HH of 2 adults and 2 children		$-$0.0197
		($$-$$1.27)
HH of 2 adults and 3 children		0.0207
		(0.92)
HH of 3 adults and children		0.00456
		(0.27)
HH of 1 adult younger than 65		0.0282*
		(1.97)
HH of 1 adult older than 65		$-$0.0166
		($$-$$1.03)
Country fixed effects	Yes	Yes
Observations	115,097	115,097

Standard errors in parentheses. $$^{*}p<0.05$$, $$^{**}p<0.01$$, $$^{***}p<0.001$$

Omitted household type is 2 adults below 65 years

Multiple imputed bootstrapped standard errors as in ECB (2020)

**Table 6:** Estimated number and percentage of vulnerable individuals in households accruing salary income (at one, three, six and twelve months) when deprived of salaries and when awarded COVID-19 layoff benefits, for a basket of food and utilities, and a basket of food and utilities, rent and mortgages

	Food and utilities				Food and utilities, rent and mortgages			
	M1	M3	M6	M12	M1	M3	M6	M12
(a) Austria								
Without salaries	7838	7838	7838	15,281	21,963	44,047	62,888	71,463
With layoff	0	0	0	5827	7312	22,073	28,994	35,222
Without salaries	0.1%	0.1%	0.1%	0.3%	0.4%	0.7%	1.1%	1.2%
With layoff	0.0%	0.0%	0.0%	0.1%	0.1%	0.4%	0.5%	0.6%
Observations	4544	4544	4544	4544	4544	4544	4544	4544
(b) Belgium								
Without salaries	78,568	150,379	286,917	491,333	235,974	712,135	1,095,138	1,757,590
With layoff	33,068	49,883	230,617	266,963	87,144	285,463	445,014	564,326
Without salaries	1.0%	2.0%	3.7%	6.4%	3.1%	9.3%	14.2%	22.8%
With layoff	0.4%	0.6%	3.0%	3.5%	1.1%	3.7%	5.8%	7.3%
Observations	3346	3346	3346	3346	3346	3346	3346	3346
(c) Germany								
Without salaries	928,515	2,113,261	4,142,438	6,707,723	2,405,772	4,532,011	7,707,629	13,269,392
With layoff	133,054	272,374	622,145	802,865	365,147	548,882	1,013,972	1,340,357
Without salaries	1.6%	3.6%	7.0%	11.3%	4.1%	7.6%	13.0%	22.4%
With layoff	0.2%	0.5%	1.0%	1.4%	0.6%	0.9%	1.7%	2.3%
Observations	7938	7938	7938	7938	7938	7938	7938	7938
(d) Finland								
Without salaries	1827	2991	3272	9416	11,342	29,557	49,917	74,849
With layoff	873	1828	2270	2270	6167	13,631	25,755	41,534
Without salaries	0.0%	0.1%	0.1%	0.3%	0.3%	0.8%	1.3%	2.0%
With layoff	0.0%	0.0%	0.1%	0.1%	0.2%	0.4%	0.7%	1.1%
Observations	18,923	18,923	18,923	18,923	18,923	18,923	18,923	18,923
(e) France								
Without salaries	104,986	215,637	396,695	729,029	869,591	2,332,955	3,733,395	5,550,351
With layoff	21,866	60,426	76,962	122,124	129,875	291,514	447,911	594,736
Without salaries	0.2%	0.5%	0.9%	1.7%	2.0%	5.5%	8.8%	13.1%
With layoff	0.1%	0.1%	0.2%	0.3%	0.3%	0.7%	1.1%	1.4%
Observations	23,052	23,052	23,052	23,052	23,052	23,052	23,052	23,052
(f) Italy								
Without salaries	4,010,568	6,743,355	9,975,795	14,452,892	5,050,221	8,599,383	12,446,091	16,533,112
With layoff	996,631	1,254,103	1,413,459	1,697,866	1,550,897	1,908,014	2,302,375	2,890,525
Without salaries	10.4%	17.5%	25.9%	37.6%	13.1%	22.4%	32.4%	43.0%
With layoff	2.6%	3.3%	3.7%	4.4%	4.0%	5.0%	6.0%	7.5%
Observations	10,915	10,915	10,915	10,915	10,915	10,915	10,915	10,915
(g) Portugal								
Without salaries	1827	2991	3272	9416	11,342	29,557	49,917	74,849
With layoff	873	1828	2270	2270	6167	13,631	25,755	41,534
Without salaries	0.0%	0.1%	0.1%	0.3%	0.3%	0.8%	1.3%	2.0%
With layoff	0.0%	0.0%	0.1%	0.1%	0.2%	0.4%	0.7%	1.1%
Observations	10,689	10,689	10,689	10,689	10,689	10,689	10,689	10,689

**Table 7:** Relative risk of informal employment

	AT	BE	DE	FI	FR	IT	PT
Rel. risk by country	1.34	1.25	1.07	2.01	1.36	1	1.22

**“Relative risk by type of employment”** is the ratio between the percentage of vulnerable individuals with temporary contract and accruing less than the minimum wage and the percentage of vulnerable individuals not meeting one or both of these conditions. Vulnerable individuals are those who cannot afford food, utilities and rents and mortgages on the main residence at the three month mark, if deprived of salaries

**Table 8:** Vulnerability at the 3 months mark using confidence intervals at 95%

(a) When individuals resort to a combination of deposits, transfers, public pensions and normal unemp. benefits.						
	Food and utilities			Food and utilities, rent and mortgages		
	Lower	Mid	Upper	Lower	Mid	Upper
AT	0.04	0.05	0.06	0.07	0.08	0.09
BE	0.09	0.11	0.14	0.15	0.18	0.21
DE	0.11	0.12	0.14	0.18	0.20	0.22
FI	0.09	0.10	0.10	0.15	0.16	0.17
IT	0.16	0.18	0.19	0.20	0.21	0.23
FR	0.08	0.09	0.10	0.17	0.18	0.19
PT	0.19	0.21	0.23	0.24	0.26	0.28
(b) For employees, considering food and utilities.						
	No salaries			With layoff		
	Lower	Mid	Upper	Lower	Mid	Upper
AT	0.0%	0.0%	0.1%	0.0%	0.0%	0.0%
BE	$-$0.1%	0.6%	1.3%	0.2%	0.2%	0.2%
DE	0.2%	0.6%	1.0%	0.0%	0.2%	0.3%
FI	0.0%	0.0%	0.0%	0.0%	0.0%	0.0%
IT	3.3%	3.3%	3.3%	0.1%	0.1%	0.1%
FR	0.1%	0.1%	0.1%	0.0%	0.0%	0.0%
PT	0.7%	1.4%	2.0%	0.1%	0.3%	0.6%
For employees, considering food and utilities and housing expenses						
	No salaries			With layoff		
	Lower	Mid	Upper	Lower	Mid	Upper
AT	$-$0.1%	0.3%	0.8%	$-$0.1%	0.1%	0.3%
BE	1.8%	3.7%	5.7%	0.3%	0.3%	0.3%
DE	0.6%	1.1%	1.6%	0.1%	0.3%	0.6%
FI	0.4%	0.4%	0.4%	0.0%	0.0%	0.0%
IT	5.0%	5.0%	5.0%	0.5%	0.5%	0.5%
FR	0.7%	0.7%	0.7%	0.1%	0.1%	0.1%
PT	3.3%	4.3%	5.3%	0.7%	1.4%	2.0%

## Footnotes

1 By privately earned income, we refer to income other than pensions and public transfers. It encompasses salary income, self-employment income, rental income, income from financial assets and regular private transfers.

2 For food expenses we consider average monthly expenditure on food and beverages at home. Utility expenses comprise average monthly expenditure on utilities, specifically electricity, water, gas, TV and internet.

3 See Browning and Crossley (2009) for further details on how households in temporarily constrained circumstances cut back on expenditures, and how they spend marginal dollars of unemployment benefits.

4 More information on the sampling design, weighting and multiple imputation, is available in ECB (2020), the ECB HFCS Wave 3 Methodological Report.

5 They take the highest value in Portugal, where they nonetheless represent only 0.6% of the population.

6 These could be households which are already resorting to atypical resources (which create financial commitments or imply for instance sales of fixed assets) to meet their most basic expenses, or, due to survey construction - where respondents report income over the last 12 months - , households who have experienced an income increase at some point over the past twelve months and have adjusted their monthly consumption upwards.

7 Data of the average length of the lockdown in each country was taken from the Data on Country Responses Measures to COVID-19 repository, elaborated by the European Center for Disease Prevention and Control: https://www.ecdc.europa.eu/en/publications-data/download-data-response-measures-covid-19.

8 We used the cash saving brackets by country provided by Esselink and Hernández (2017) to decide on amounts allocated. Cash savings were allocated randomly among individuals aged 18 or above, in percentage of the countries’ cash holders. Individuals randomly selected as storing savings in cash were then randomly assigned to a bracket of precautionary savings in cash, replicating country percentages, and allocated the midpoint of the interval. Individuals assigned to the bracket “above 1000” were assigned 1500 euros.

9 In a regression analogous to column (2) of Table 5, but restricted to households with two adults (with or without children) with at least one earner

10 In addition, Table 6 contains our results for 1, 3, 6 and 12 months.
